# Relative shortening and functional tethering of spinal cord in adolescent scoliosis – Result of asynchronous neuro-osseous growth, summary of an electronic focus group debate of the IBSE

**DOI:** 10.1186/1748-7161-3-8

**Published:** 2008-06-27

**Authors:** Winnie CW Chu, Wynnie MW Lam, Bobby KW Ng, Lam Tze-ping, Kwong-man Lee, Xia Guo, Jack CY Cheng, R Geoffrey Burwell, Peter H Dangerfield, Tim Jaspan

**Affiliations:** 1 Department of Diagnostic Radiology and Organ Imaging, The Chinese University of Hong Kong, Prince of Wales Hospital, Shatin, Hong Kong, PR China; 2 Orthopaedics and Traumatology, The Chinese University of Hong Kong, Prince of Wales Hospital, Shatin, Hong Kong, PR China; 3 Department of Rehabilitation Sciences, The Hong Kong Polytechnic University, Hong Kong, PR China; 4 Orthopaedics and Traumatology, The Chinese University of Hong Kong, Prince of Wales Hospital, Shatin, Hong Kong, PR China; 5 The Centre for Spinal Studies & Surgery, Nottingham University Hospitals Trust, Queen's Medical Centre Campus, Nottingham NG7 2UH, UK; 6 Sherrington Buildings, Ashton Street, Liverpool, L69 3GE, UK; 7 Department of Radiology, Nottingham University Hospitals Trust, Queen's Medical Centre Campus, Nottingham NG7 2UH, UK

## Abstract

There is no generally accepted scientific theory for the causes of adolescent idiopathic scoliosis (AIS). As part of its mission to widen understanding of scoliosis etiology, the International Federated Body on Scoliosis Etiology (IBSE) introduced the electronic focus group (EFG) as a means of increasing debate on knowledge of important topics. This has been designated as an on-line Delphi discussion. The Statement for this debate was written by Dr WCW Chu and colleagues who examine the spinal cord to vertebral growth interaction during adolescence in scoliosis. Using the multi-planar reconstruction technique of magnetic resonance imaging they investigated the relative length of spinal cord to vertebral column including ratios in 28 girls with AIS (mainly thoracic or double major curves) and 14 age-matched normal girls. Also evaluated were cerebellar tonsillar position, somatosensory evoked potentials (SSEPs), and clinical neurological examination. In severe AIS compared with normal controls, the vertebral column is significantly longer without detectable spinal cord lengthening. They speculate that anterior spinal column overgrowth relative to a normal length spinal cord exerts a stretching tethering force between the two ends, cranially and caudally leading to the initiation and progression of thoracic AIS. They support and develop the Roth-Porter concept of *uncoupled neuro-osseous growth *in the pathogenesis of AIS which now they prefer to term '*asynchronous neuro-osseous growth'*. Morphological evidence about the curve apex suggests that the spinal cord is also affected, and a *'double pathology' *is suggested. AIS is viewed as a disorder with a wide spectrum and a common neuroanatomical abnormality namely, a spinal cord of normal length but short relative to an abnormally lengthened anterior vertebral column. Neuroanatomical changes and/or abnormal neural function may be expressed only in severe cases. This *asynchronous neuro-osseous growth concept *is regarded as one component of a larger concept. The other component relates to the brain and cranium of AIS subjects because abnormalities have been found in brain (infratentorial and supratentorial) and skull (vault and base). The possible relevance of systemic melatonin-signaling pathway dysfunction, platelet calmodulin levels and putative vertebral vascular biology to the *asynchronous neuro-osseous growth concept *is discussed. A biomechanical model to test the spinal component of the concept is in hand. There is no published research on the biomechanical properties of the spinal cord for scoliosis specimens. Such research on normal spinal cords includes movements (kinematics), stress-strain responses to uniaxial loading, and anterior forces created by the stretched cord in forward flexion that may alter sagittal spinal shape during adolescent growth. The *asynchronous neuro-osseous growth concept *for the spine evokes controversy. Dr Chu and colleagues respond to five other concepts of pathogenesis for AIS and suggest that *relative anterior spinal overgrowth *and *biomechanical growth modulation *may also contribute to AIS pathogenesis.

## Background

In the absence of any accepted scientific theory for the etiology of idiopathic scoliosis treatment remains pragmatic with a very incomplete scientific basis. The International Federated Body on Scoliosis Etiology (IBSE) introduced the electronic focus group (EFG) as a means of increasing debate on knowledge of important topics. The Statement written by Dr WCW Chu and colleagues is based on important spinal imaging research, neurological findings and anthropometric data already published [1-3], Moderator see [4]. The research is part of a series of studies [5-11] to evaluate the hypothesis that in AIS there is a systemic disturbance of growth [12-15] manifest in each of the appendicular skeleton, vertebral column and skull that points to a problem of axial skeletal growth control [7]. In addition to skeletal findings, neurological features are also addressed including clinical neurological examination, cerebellar tonsillar level at the foramen magnum and somatosensory evoked potentials (SSEPs). They address the pathogenesis and pathomechanisms of AIS, not its etiology.

**Figure 1:**
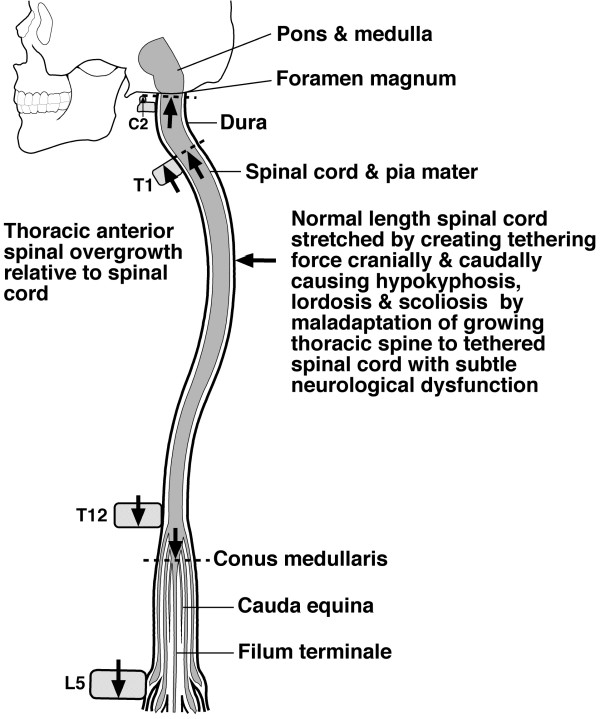
*Uncoupled, or asynchronous, neuro-osseous growth concept for AIS pathogenesis *involving asynchronous growth of spine and spinal cord. Diagrammatic representation from the findings of Chu et al [1,2]. showing how anterior spinal overgrowth (T1-T12) stretches the normal length spinal cord cranially and at the cauda equina leading to hypokyphosis (horizontal arrow ?from anterior component of force [42]) with maladaptation of the growing thoracic spine to the tethered spinal cord which, with subtle neurologic dysfunction, forms a scoliosis. Diagram modified from Breig [43]. *Uncoupled, or asynchronous, neuro-osseous growth concept for AIS pathogenesis *involving asynchronous growth of spine and spinal cord. Diagrammatic representation from the findings of Chu et al [1-4] showing how anterior spinal overgrowth (T1-T12) stretches the normal length spinal cord cranially and at the cauda equina leading to hypokyphosis from an anterior component of force (horizontal arrow [16]) with maladaptation of the growing thoracic spine to the tethered spinal cord which, with subtle neurologic dysfunction, forms a scoliosis. Diagram modified from Breig [17].

Dr Chu applied the new method of *multiplanar reformat magnetic resonance imaging *to the spine of 28 AIS girls and 14 matched normal girls [1]. In severe AIS compared with normal subjects, the thoracic vertebral column is significantly longer without detectable change in spinal cord length evaluated as *cord-to-vertebral length ratios *(Figure 1). They speculate that the initiation and progression of AIS result from vertebral column overgrowth through a lordoscoliotic maladaptation of the spine to the subclinical tether of a relatively short spinal cord. This interpretation accommodates both the lordotic [18-21] and the dorsal shear force [22] concepts for the pathogenesis of AIS.

Their research confirms and extends the speculation of Roth [23-25] and Porter [26-30] that *disproportion of vertebro-neural growth *[23-25] and *uncoupled neuro-osseous growth *[28] explains the pathogenesis of progressive AIS. There is other morphological evidence that supports Dr Chu's view that the *neuraxis *in idiopathic scoliosis may be under tension in the axial direction [28,31]. During the course of this debate Dr Chu and colleagues preferred the term *asynchronous neuro-osseous growth *[3], for while the evidence suggests that the pace, or velocity, of growth differs in the vertebral column and spinal cord, it is unknown whether, or how, the vertebral and cord growth are *'coupled' *before the onset of AIS.

### Neurological features

Previous research in Hong Kong introduced a redefined MRI reference level at the foramen magnum (at and below the basion-opisthion line) to diagnose asymptomatic Chiari 1 malformation [[32], Moderator see [33]]. In 164 patients with AIS, tonsillar ectopia was found in 33% of patients with abnormal SSEPs and 2.9% of patients with normal SSEPs [34] pointing to a neural origin. In AIS, tonsillar ectopia significantly different from normal is established [35,36]. Abnormal SSEPs were found in 17/147 (11%) of AIS patients [[37], see [38]]. SSEPs reflect the presence of disturbed standing balance control when the subject relies on somatosensory input [39] with functional delay above the cervical level in most patients [40]. But there is evidence that SSEPs do not correlate significantly with Cobb angle changes after one year of rehabilitation [41]. The *conus medullaris *is at a normal position [42] in AIS [43], Dr Chu and colleagues speculate that 1) subjects with severe scoliosis could have subclinical cord tethering without clinically detectable neurological deficit, and 2) AIS is a disorder of wide spectrum with neuroanatomical changes and/or abnormal neural function only emerging with severe scoliosis.

### A double pathology?

**Figure 2:**
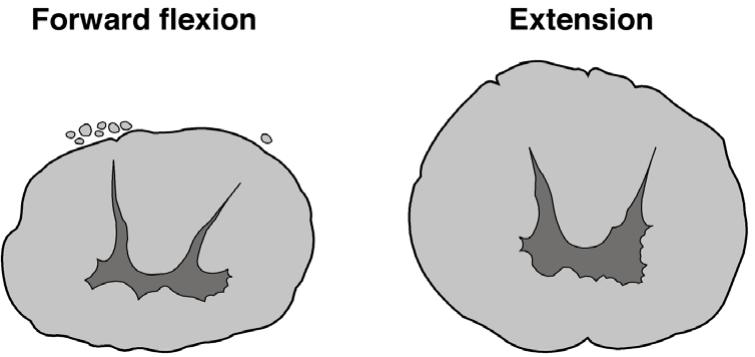
Diagrams from transverse sections through the spinal cords at T3 of cadavers in forward flexion and extension showing the rounder shape of the cord in extension. Chu et al [2] describe a similar difference in apical spinal cord shape between normal and thoracic AIS subjects measured as the ratio of antero-posterior (AP) to transverse (TS) diameter of the cord (see Response to Comment 10). Diagrams drawn from Breig [43]. Diagrams from transverse sections through spinal cords at T3 of two cadavers in forward flexion and extension showing the rounder shape of the cord in extension. Chu et al [2-4] describe a similar difference in apical spinal cord shape between thoracic AIS and normal subjects measured as the ratio of antero-posterior (AP) to transverse (TS) diameter of the cord (see Response to Comment 10). Diagrams drawn from specimens reported by Breig [17].

Not only is linear vertebral column growth abnormally increased in AIS, but Dr Chu and colleagues report morphological evidence [2-4] of altered spinal cord shape about the curve apex suggesting that the cord is also affected ie a *'double pathology' *may exist involving spine and cord (Figure 2). Consideration is required for the cause of each of 1) vertebral column overgrowth, and 2) putative impaired spinal cord growth. Dr Chu and colleagues address these aspects further in recent papers [3,4]. The *vertebral overgrowth *of AIS may be explained by the concept of *primary skeletal change *[44] as it affects the sagittal plane of the spine with anterior increments and posterior decrements as pathogenesis and, as histogenesis,*uncoupled, or asynchronous, endochondral-membranous bone growth *[6,7]. Roth explained AIS pathogenesis as an exaggeration of the normal differential growth of the cord and spine by hormonal abnormality [*'vertebro-neural growth theory*', [23-25,45]]. Porter [28] stated that the *putative impaired growth of the spinal cord *in AIS may result from an abnormal response to stretch including: 1) inadequate cord growth from deficient hormonal environment; 2) cell membrane defect with abnormal function of contractile proteins in cells of the spinal cord as part of a systemic disorder; 3) abnormality of the elastin fibre system, 4) failure of melatonin to scavenge free radicals resulting in spinal cord stretch injury; and 5) hypoxia.

### Biomechaniccs of the central nervous system and pathologic axial tension

There is a lack of research on the biomechanical properties of the spinal cord in scoliosis specimens. Dr Chu and colleagues have a biomechanical model in hand to test the asynchronous neuro-osseous growth concept of AIS. There is research on the biomechanical properties of the normal spinal cord in relation *in vivo *kinematics caused by spinal movements [16,17,46-50], viscoelastic properties [51-56] and, some evidence for the development of an *anterior component of force *in spinal forward flexion [16]; the latter which may be relevant to scoliosis pathogenesis is not debated in this EFG.

From the biomechanical standpoint the *continuous axial neural tract (neuraxis) *of pons, medulla oblongata, spinal cord and cauda equina is a functional unit between mesencephalon and lumbo-sacral ganglia [17,47]. When the normal adult spinal canal is elongated by forward flexion, the *neuraxis *is axially stretched between its cranial and caudal points of fixation and, when backward extended, is slackened [16,17,46-49]. Breig [17] examined cadavers and living subjects and found that in *spinal flexion/extension *the spinal cord: 1) unfolds and folds; 2) does not move up and down axially in the spinal canal but adapts to the varying length of the canal by *plastic deformation *– lengthening in flexion and shortening in extension; 3) cross-sectional area decreases in flexion and increases in extension (Figure 2); and 4) resistance to further cord elongation in pronounced flexion is taken up primarily by pia mater and to a lesser extent by cord substance. In *lateral flexion*, both spinal canal and *neuraxis *elongate on the convexity and shorten on the concavity. During spinal flexion, or lateral flexion, there is slight elastic tension with cord lengthening limited by an unyielding pia mater. Breig [17] suggested that processes which increase axial tension in the neuraxis namely, *changes in relative lengths of spinal canal and cord*, can lead to *pathologic axial tension *and damage the cord and spinal nerves by overstretching. In health, cord function, synaptic transmission, and metabolism all continue undisturbed within dynamically changing cord tissue [46,50].

### Spinal cord kinematics and the anterior component of force in flexion

In monkeys [46], the cervical cord in flexion stretches most (24%) and least in the caudal cord (4%). Below the mid-cervical level, movements in flexion are toward the head and above this level are away from it. In human cadavers, Reid [16] found that the length of the spinal canal increased up to 18 mm at C8-T5 roots on forward flexion. In AIS girls the mean sitting height lengthening is 20 mm [13]. In the thoracic kyphosis of human cadavers in the normal erect posture, the cord and dura (theca) are closely apposed to the anterior wall of the spinal canal and loosely tied by fibrous bands to the posterior longitudinal ligament [16]. In different degrees of spinal flexion, Reid [16] measured the *anterior component of a force *exerted by the cord and dura at different levels (C3-T12) and found *physiological lengthening *of cord and dura, chiefly between C2-T1 up to a maximum of 17.6%; and the *anterior component of the force *which reached maximum values of 30–40 lb/sq inch (207–276 kilopascals) (Figures 1 &2).

These data suggest a mechanism for thoracic AIS by which the *hypokyphosis *about the curve apex may come about. A spinal cord of normal length stretched by linear vertebral overgrowth may create an *anterior component of force *that exerts pressure on, and flattens, the kyphos. Mid-thoracic vertebrae have a pre-existing rotation to the right [57]. In Dr Chu's concept for AIS the lateral spinal curve and axial vertebral rotation evolve together, by the cord tether restraining linear vertebral overgrowth – as in the *spring model *of Roth [24,25]. Scoliosis evolution with its lateral spinal flexion – by analogy with Reid's [16] findings of cord lengthening in forward flexion – should lead to *structural lengthening *of cord and dura, despite the move to a posterior centre of axial rotation [26,58]. This *lengthening of the cord in a scoliotic *spine suggests that where Dr Chu and colleagues *measured the cord length as normal *in the deformed spine, *before *curve initiation the cord was *short in absolute terms*. In normal children, asynchronous growth of cord and column in early adolescence may contribute to the flattening of the kyphosis in normal girls [59,60]. In progressive infantile idiopathic scoliosis, the cord axial rotation may not always adjust to the change in anatomy of the vertebral column [61], an observation confirmed for AIS [31].

### Visco-elastic properties of spinal cord

The biomechanical properties of the human cadaveric spinal cord within physiological levels of loading have been studied under axial tension and longitudinal elongation in isolated specimens freed from dura mater [53,54]. Uniaxial tension applied at moderate strain rates showed a non-linear stress-strain response with increasing strain increasing the tangent modulus; and a significant relaxation over 1 minute [53]. The pia mater gives the isolated human spinal cord its tensile strength in the axial direction [54]. In animals, uniaxial biomechanical properties of the spinal cord have been reported for anesthetised puppies [51] and cats [52], cattle [56] and rats [55]. The rat spinal cord subjected to moderate strain rates showed non-linear viscoelastic characteristics commonly seen with biological tissues, fitted to an integral non-linear viscoelastic model [55]. We are aware of no studies on the mechanical properties of the spinal cord specimens from either experimental scoliosis [62,63], or scoliotic subjects. One difficulty with human spinal cords if and when available is the rapid deterioration in the quality of the tissue after excision [56].

### Neck flexion

Restriction of neck flexion with AIS has not been reported except for a special group of boys [64,65]: twelve adolescent males had mild thoracic scoliosis of unknown pathogenesis and markedly restricted neck flexion with no other anomalies detected, and described as a distinct type of adolescent "idiopathic" scoliosis. The most prominent feature in 9/12 subjects was the presence of mild thoracic or neck pain. To explain the thoracic scoliosis, limited neck flexion, and pain, local or distant cord tethering [64] and a short spinal cord [65] are suggested. Neck flexion/extension alters spinal cord shape [17] (Figure 2).

### Neuro-osseous growth involving the head as well as the spine

The asynchronous neuro-osseous growth concept of Dr Chu and colleagues does not explain higher level *CNS *disturbances in AIS including vestibular function [66-70]. This led Dr Chu and colleagues to scan the brain, search for and find 1) volumetric regional brain volume differences between AIS subjects and normal controls [[71,72] Moderator see [73,74]], and 2) abnormalities of the skull base [8,9] and vault [10]. This EFG debates the brain asymmetries, a possible primary deficit of interhemispheric coordination, skull abnormalities (of vault and base) and the larger foramen magnum [35]. It raises questions about the embryology, particularly of neural crest [75], and the evolution of the normal human gracile skull and possibly gracile human spine in relation to the inactivated *MYH16 *gene [76].

*The research *engenders controversy, including:

1. Why don't all children with cord tethering develop scoliosis?

2. A cause-and-effect relationship has not been demonstrated, only an association.

3. Should thoracic AIS be caused by spinal cord tethering of the adjacent growing spine, how can cord tethering initiate thoracolumbar and lumbar AIS?

4. If the neural axis tethers the anterior spinal column, how can this determine the laterality patterns of AIS?

5. Cord tethering in syringomyelia does not cause a predominant laterality for the associated scoliosis [77], so how can a short spinal cord explain the laterality patterns of AIS?

6. Why neck flexion is not restricted in thoracic AIS apart from a distinct type of adolescent 'idiopathic' scoliosis in males [64,65]. A neuraxis under *pathologic axial tension *in AIS should limit spinal cord elongation in forward flexion [16,17,47-49] which evidently it does not.

7. Mechanical properties of spinal cord relative to physiological forces created by traction from linear vertebral growth [51-56].

8. Can the '*double pathology' concept *be validated in a biomechanical test model?

9. Whether the altered apical spinal cord shape of thoracic AIS results from an abnormal spinal cord or, is secondary to the apical hypokyphosis with its local structural spinal extension (Figure 2).

9. How to account for extra-spinal left-right skeletal length asymmetries [13-15,78-83] and disproportions by *asychronous neuro-osseous growth*

10. Speculation that a process producing left-right spinal asymmetry (? from end-plate physes, osseous, discal, or neuromuscular tissues) together with rapid spinal elongation, brain maturation delay, upright posture and trunk movements [84] may contribute to the initiation and progression of AIS [[85], Moderator see [86,87]].

11. Might the results of applying gadolinium-enhanced MR imaging [88-90] to vertebral growth plates in AIS shed light on the mechanism of any uncoupled, asynchronous, neuro-osseous growth?

12. Whether spinal nerve roots may develop *'insufficiency' *and, with it, left-right asymmetries to induce spinal root neuro-osseous tethers [[23,91] Moderator see [92]].

13. Neural abnormalities for a minority of AIS groups being used to interpret the findings for the majority.

14. Does the lower position of cerebellar tonsils result from spinal cord tether? Or, in some subjects, from a relatively small posterior cranial fossa (as in Chiari I malformation) leading to overcrowding of the hind brain causing the cerebellar tonsils to be pushed down? [93].

15. The view that pontine and hindbrain regions are the likely sites of primary pathology that lead to idiopathic scoliosis [94].

16. The concept does not explain higher level *CNS *disturbances including vestibular dysfunction [66-74].

17. Anecdotal evidence that prolonged rest in bed halts the progress of scoliosis [95,96].

18. Whether different mechanisms are involved in the initiation and progression of AIS [15,21,67,97-104].

19. Whether systemic melatonin-signaling pathway dysfunction in AIS [105-111] may affect the spinal cord and meninges.

20. Five other concepts that attempt to explain the pathogenesis for AIS are debated: a) relative anterior spinal overgrowth [6,7,18-21]; b) right thoracic curves in adolescent girls as a pediatric nosological entity [80,112-114] involving the autonomic nervous system and expressed through the ribcage [Moderator see [92,115];] c) scoliosis as a biomechanical abnormality arising in hip movement asymmetry [[116-121], Moderator see [122,123]]; d) a neurodevelopmental concept [[85], Moderator see [86,87]]; and e) biomechanical spinal growth modulation [[101-103], Moderator see [104,124]].

21. Whether the concept of subclinical cord tethering has relevance to the pathogenesis of non-idiopathic scoliosis [29] and scoliosis associated with abnormal *CNS *findings [[93,125,126], Moderator see [127]].

In summation, this EFG aims to explore what may be learnt about the pathogenesis of AIS by IBSE members debating via e-mail the findings of Dr Chu and colleagues revealed by applying a new MR imaging technique to progressive adolescent idiopathic scoliosis [Moderator see [128]]. Clinical, biological and biomechanical issues relating to the *concept of uncoupled, or asynchronous, neuro-osseous growth *in the initiation and progression of AIS are discussed with new concepts emerging. Recent relevant research on AIS pathogenesis is considered.

## Statement by Dr Chu and colleagues

We undertook a comparative study of magnetic resonance (MR) imaging of the whole vertebral column and spinal cord in girls with adolescent idiopathic thoracic scoliosis (AIS) and age and gender matched normal subjects [1]. The aim of the study was to investigate the relative length of spinal cord to vertebral column in AIS and to correlate with cerebellar tonsil level and somatosensory evoked potentials (SSEPs)(Figure 1).

14 AIS girls with mild to moderate curve (<30 degrees) and 14 AIS girls with progressive curve (> 40 degrees) were compared with 14 normal girls aged 13–14 years. Using the multi-planar reconstruction technique, the length of the vertebral column (from tip of C2 down to the inferior end plate of L5) and the length of spinal cord (from tip of C2 down to the conus medullaris) were measured. Compared with normal controls, the vertebral column is significantly longer in severe AIS. The latter was contributed to mainly by lengthening of the thoracic segment of the vertebral column. But, the absolute spinal cord length did not show corresponding lengthening in AIS. Therefore, the ratio of cord length/vertebral length was significantly reduced in severe AIS (ratio = 0.67) compared with the normal controls (ratio = 0.72).

The concept of uncoupled neuro-osseous growth has been suggested in the pathogenesis of AIS [23-28]. According to this concept, longitudinal growth of the spinal cord fails to keep pace with growth of the vertebral column, and the idiopathic scoliosis is a consequence of maladaptation of the growing immature spine to a tether created by a short spinal cord. The findings of our current study support the above hypothesis. A significantly lower cerebellar tonsil level (28% of all AIS) and abnormal SSEP findings (43% of severe AIS and 14% of mild AIS) were found in these subjects when compared with the controls. Similar findings have been observed in our previous papers [34,37].

We postulate that the increased prevalence of tonsillar ectopia [32,34], syringomyelia [127,129-131], abnormal SSEPs [34,37,38] and abnormal postural balance [39] in AIS are not isolated co-incidences but represent clinical manifestations of a common neuro-anatomical abnormality, which could be explained by the relative shorter cord in AIS. As the brain inside the cranium is fixed while the lower nerve roots exit normally via the neural foramina in the lengthened vertebral column, we speculate that the relative shorter cord was under a stretching tethering force from the two ends, cranially and caudally. Such tethering could affect anterior spinal column growth leading to a progressive hypokyphosis and lordosis of the thoracic spine which coupled with the subtle neurological dysfunction can then initiate the process of lordoscoliosis deformity in the thoracic spine [29]. This is a preliminary study. We are working on a larger number of patients and planning a longitudinal study involving analyses between multiple variables (Moderator: Dr Chu and colleagues have recently published their findings for spinal cord morphology in 97 AIS girls, and 36 age-matched controls [4]).

## General Statements

### Comment no. 1

The observations relate to lengths of the vertebral column and spinal cord in two groups of so-called idiopathic scoliosis, 14 girls AIS (<30 degrees) and 14 girls AIS (>40 degrees) in comparison with 14 normal girls are good and proper. The article is very interesting.

### Comment no. 2

The authors present the results of a morphometric MRI study of the spine in right thoracic AIS in girls (Rcx-T-F-AS) using the multiplanar reconstruction MRI technique. It is the second of two MRI studies on thoracic AIS from that centre. Guo et al [6,7] had used MRI vertebral morphometry of the thoracic spine in AIS which was stated to confirm and support the consensus of the relatively faster growth of anterior than posterior elements of the thoracic vertebrae. In both studies [1,6] different parameters of vertebrae have been measured and compared between scoliotic and normal subjects.

#### Responses

In the current study [1], we have employed the new imaging sequence of multiplanar reformat technique so that the scoliotic spine can be reconstructed into a straightened best mid-sagittal section of the vertebral column and spinal cord. The whole length of each structure can therefore be measured on a single plane while in the previous study [6] multiple sagittal planes were taken to ensure true sagittal representation of different parts of the curvature of the spine. In conclusion, the new measurement parameters undertaken by the current study is not feasible by using the previous MR technique.

### Comment no. 3

Dr Chu and colleagues

a) Support the concept of uncoupled neuro-osseous growth, namely that "...longitudinal growth of the spinal cord fails to keep pace with growth of the vertebral column."

b) Support the concept that idiopathic scoliosis is a consequence of maladaptation of the growing immature spine to a tether created by a relatively short spinal cord.

c) Speculate that relative low position of the cerebellar tonsil is related to relative shortening and functional tethering of the spinal cord.

d) Suggest that patients with severe scoliosis could have subclinical cord tethering.

## Curve types and abnormal skeletal growth pattern

### Comment no. 4

The AIS study population comprises 14 girls with mild scoliosis (<30 degrees Cobb angle) and 14 girls with severe scoliosis >40 degrees Cobb angle).

a) What are the curve types?

b) What is the reproducibility of measuring cerebellar tonsillar ectopia?

#### Response

a) The curve types are as follows: 14 girls with mild scoliosis (7 with Lenke type I, 7 with Lenke type III) and the 14 girls with severe scoliosis (6 with Lenke type I, 1 with Lenke type II and 7 with Lenke type III).

b) In the previous study [32], the intraclass correlation coefficient was 0.996 indicating a very high inter-observer reliability for the measurement.

### Comment no. 5

There is controversy on methods to analyse stature and sitting height [13,132]. Standing height is reported as uncorrected for the loss of height due to the lateral spinal curvature.

a) Do you have figures for corrected heights (standing and sitting) for the AIS girls with mild and severe curves?

b) If not, can the corrected heights be calculated from the data generated by the multiplanar reconstruction MRI technique?

#### Response

a) The mean corrected standing heights for the subjects are calculated with the Bjure-Nachemson Formula [133]. The corrected height = uncorrected height + y, where log y = 0.011x-0.177 and x = Cobb angle. The results are as follows: severe AIS = 159.4 cm, mild AIS = 159.2 cm and in normal controls = 155.1 cm.

b) Theoretically, corrected height can be estimated by the difference between the longitudinal height of subject's body and the entire length of the vertebral column measured by multiplanar reformat MRI.

### Comment no. 6

Dr Chu and colleagues explain the localization of anterior column lengthening to the thoracic vertebrae by growth plate closure later than in the cervical and lumbar spines, quoting Ganey and Ogden [134]. But this quote relates to the *neurocentral synchondroses closure *with the last closure about 6–7 years and not the *vertebral endplate physeal closure *which determines the relative anterior spinal overgrowth during adolescence.

#### Response

Thank you for pointing out the misinterpretation. Lord and Ogden et at [135] stated that there are definite, contiguous, plate-like physes superiorly and inferiorly, comparable to the proximal and distal physes of any long bone that allow the vertebral body to increase in height. Because growth is limited, cell column formation is neither as long nor as organized as in long bone physes. There are however, no reported data on the exact timing of fusion in these physes. Most studies evaluated the fusion only of neurocentral synchondroses.

## Earlier radiological morphometric studies

### Comment no. 7

After the CT scanning method was applied to the scoliotic spine [136,137] in the early 1990s the principles were applied in a series of morphometric studies using different skeletal parameters of scoliotic and normal vertebrae published by a Swedish group [138-142].

a) In what respect is the multiplanar reconstruction MR method superior to the earlier morphometric radiological studies?

b) Were they any important differences between the results of studied parameters obtained by the use of the two methods?

#### Response

a) We think the major advantage of MR method is lack of radiation when compared with CT. This is particularly important for reducing the hazard of breast cancer risk in adolescent girls.

b) We have looked into the vertebral morphometry of apical vertebrae in AIS subjects and compared with normal controls (results presented in Chu et al [2] using MR multiplanar reformat technique [Moderator see [3,4]]). We have found that there is exaggerated asymmetry of the neural arch in AIS subjects with smaller pedicle width, length and area on the concavity. The results are in agreement with findings by Xiong et al [140].

### Comment no. 8

#### Sagittal wedge angle of vertebrae

We studied the sagittal configuration of the posterior elements in early thoracic scoliosis. A significant decrease of the *sagittal wedge angle *of the apical and the vertebra subjacent to it – both united by the same rib – was first registered in curves with coronal Cobb angle of 8–15 degrees. *All five vertebrae *of the apical segment were significantly wedged in curves of not less than 16–30 degrees [139].

These observations while consistent with a pathogenetic concept for AIS of an anterior-posterior vertebral growth disproportion from either biological or mechanical (spinal cord) cause, they do not allow any conclusion to explain the initiating cause of the decreased sagittal wedging of the thoracic vertebrae, ie the etiology of the spinal deformity.

#### Response

We think that the anterior relative overgrowth of the vertebra coupled with the relative slower growth of the spinal cord produce a coupling effect manifested maximally at the apical region of the thoracic scoliosis cases as a decrease in the sagittal wedge angle. Our previous MRI morphometry of thoracic vertebrae also showed the maximum effect at the apical and adjacent vertebrae.

## Uncoupled, or asynchronous, neuro-osseous growth concept

### Comment no. 9

The *uncoupled, or asynchronous, neuro-osseous growth hypothesis for AIS *as postulated by Dr Chu and colleagues has several requirements (Figure 1 and reference [1] Figure 6]:

1) Appendicular skeleton – taller standing height (corrected), sitting height (corrected), arm span, sub-ischial height and body mass index.

2) Vertebral column – relative overgrowth of thoracic anterior spinal column.

3) Spinal cord – normal length (but shortening relative to spine).

4) Tethering of cord anatomically.- low lying tonsil, Chiari I, syringomyelia.

5) Tethering of cord functionally – abnormal neurology.

Do you consider the vertebral and extra-spinal skeletal length overgrowth, apparently seamless, to have a common origin?

#### Response

We think that the vertebral and extra-spinal skeletal length overgrowth are governed by the same underlying mechanism of abnormal skeletal growth and abnormal spinal growth is a part of the whole systemic growth disturbance. We have demonstrated abnormal appendicular skeletal growth and osteopenia in AIS subjects in our previous studies [5,143-147].

### Comment no. 10

#### CNS and stretch from vertebral growth

In view of evidence for *accelerated vertebral growth in AIS subjects *Dr Chu and colleagues pose two questions (Figure 2):

1) Will spinal cord growth keep pace with the vertebral column growth?

2) If not, will the central nervous system be affected? *Is there a dual spinal pathology? *Theoretical argument suggests that for uncoupled neuro-osseous growth to induce AIS a *double spinal abnormality *is needed, viz:

a) Vertebral growth (abnormally increased in AIS) may stretch a *normal *spinal cord without evident damage.

*b) If the cord does not stretch normally *a scoliosis could be induced – through mechanisms outlined by Porter [28].

c) Such cord *'understretch' *would require an abnormal spinal cord.

d) If so a *double pathology *involving vertebrae and cord is required.

e) Can the latter hypothesis be tested directly?

f) If not, the uncoupled neuro-osseous concept for AIS is speculative and indirect approaches are needed to test it.

#### Response

In a recent study [2,3], we found that the ratio of antero-posterior (AP) to transverse (TS) diameter of the cord was increased in AIS patients and correlated negatively with cord-to-vertebral length ratio (Moderator see [4]). The cord was markedly deviated to the concavity at the apical level with significantly increased lateral cord space ratio, which correlated negatively with cord-to-vertebral length ratio. Our study suggests the presence of tethering and increased tension along the longitudinal axis of spinal cord results in morphological changes of cross-sectional shape and relative position of the cord – an observation which is particularly obvious in the apical region In conclusion, there is indirect evidence that the cord in AIS is "abnormal" and we hypothesize that both vertebral column and spinal cord are affected in AIS (ie.'double pathology' exists) (Moderator, spinal forward flexion and extension affect spinal cord shape (Figure 2) as may the apical hypokyphosis of thoracic AIS).

### Comment no. 11

#### MRI findings and neural abnormalities

The authors postulate that the increased prevalence of tonsillar ectopia, syringomyelia, abnormal SSEPs and abnormal postural balance in AIS are clinical manifestations of a common neuroanatomical abnormality all explained by a relatively shorter spinal cord in AIS. Dr Chu and colleagues speculate that the relatively shorter cord is under a stretching force from both ends, cranially and caudally. Such tethering could affect the anterior spinal column leading to a progressive hypokyphosis and coupled with subtle neurological dysfunction can initiate the deformity of AIS.

The *statistical *findings for the 28 AIS patients (14 mild, 14 severe curves) are:

1) Vertebral length in thoracic, but not cervical or lumbar spine, is detectably longer than normal but only for the severe scoliosis group.

2) Spinal cord length is not detectably different in the three groups, mild, and severe scoliosis, and normal.

3) The ratio of total spine length/total spinal cord length is increased but only in the severe group.

4) Cerebellar tonsillar tip not ectopic – 4/14 (28.6%) of the mild and severe scoliosis group – had the tonsillar tip below the BO line (are these figures correct?). Is the ectopia symmetrical?

5) SSEPs abnormal in 2/14 mild scoliosis and 6/14 severe scoliosis group – are these statistically significant?

6) Subtle neurological deficit in some patients with AIS. How many were affected in the two groups?

Little or no neural abnormality was detected in the mild scolioses. Neural changes were detected mainly in the more severe scolioses but overall possibly in less than 50% of those subjects. The percentages show that the tonsillar tip was not abnormal in 71% and SSEPs in 79%. In Dr Chu's full paper [1] it is suggested that scoliosis is a disorder with a wide spectrum, i.e.with and without detectable neural change.

a) The neural abnormalities for a minority in the AIS groups are being used to interpret the findings for the majority. Is this appropriate?

b) What percentage of each of mild and severe scoliosis have a composite neural abnormality – i.e. tonsillar ectopia, abnormal SSEPs and abnormal clinical neurologic signs?

c) What is the evidence that tonsillar ectopia and positive SSEPs are related to the scoliosis?

d) What are you views on possible lipid peroxidation of neuronal membranes in relation to AIS? [28,148].

#### Response

4) The patients with mild scoliosis and severe scoliosis each had 4/14 with the tonsillar tips below the BO line. The low-lying tonsils are fairly symmetrical.

5) Statistically significant (Chi square = 8.6471, p = 0.0133, df = 2).

6) Subtle neurological deficits have been reported in other cohorts. We have not performed sophisticated neurological tests on our subjects.

a) Though neural abnormalities have been reported in a relatively small percentage in different cohorts, the reported abnormalities are consistent and have been observed in many centres. As we have pointed out that AIS is probably a disorder of wide spectrum, abnormal neural function or anatomic changes might only emerge in the most severe cases. However these clinical manifestations still provide us with clues for the possible etiology of this disorder.

b) In this cohort, three out of 28 subjects (11%) had both low-lying tonsils and abnormal SSEP findings.

c) Tonsillar ectopia and SSEP abnormalities are not observed in the normal controls of our cohort, while reported in our previous published study [34], there was significant association of tonsillar ectopia and abnormal somatosensory function in AIS patients with severe curve.

d) This is an interesting speculation that the current MRI study would not be able to contribute.

### Comment no. 12

In connection with asymptomatic Chiari I malformation and AIS, Sun et al [36,149] used MRI to examine 205 AIS patients with a Cobb angle above 40 degrees and 86 age-matched healthy adolescents all of whom were neurologically normal on physical examination. The prevalence of tonsillar ectopia in AIS subjects (0 to 5.2 mm below the BO line) was higher than in healthy adolescents (0 to 1.8 mm). No significant correlations were found between cerebellar tonsil position and age or sex in AIS or control subjects. In AIS patients, cerebellar tonsil position was not statistically significant by curve severity or pattern except for double thoracic curves which had a greater prevalence of tonsillar ectopia. It was concluded that a lower cerebellar tonsil position may play an important role in pathogenesis but not in the development of AIS. Tonsillar ectopia of 2 mm or more in AIS subjects was regarded as abnormal.

#### Response

The above findings agree with the observations from our group [1,32,34,35]. The tonsillar ectopia is probably related to a relative shortened and tethered spinal cord. As the brain inside the cranium is fixed while the lower nerve roots exit normally via the neural foraminae in the lengthened vertebral column, we speculate the relative shorter cord is under a stretching tethering force from the two ends, both cranially and caudally. This might explain why the prevalence of low-lying tonsils was increased in AIS patients with marked scoliotic curves.

### Comment no. 13

The *uncoupled, or asynchronous, neuro-osseous growth concept *accommodates -

a) the general skeletal overgrowth abnormality as applied to the spine,

b) the left-right asymmetries of the appendicular skeleton, and

c) speculates that a spinal cord abnormality with growth *'insufficiency' *leads to *'a stretching tethering force from the two ends, both cranially and caudally' *which causes the initiation and progression of thoracic AIS (see Comment no. 12, Response).

To explain with AIS each of the lower position of cerebellar tonsils an alternative view is that some subjects may have a relatively small posterior cranial fossa (*PCF*) with normal-sized hind brain leading to hind brain overcrowding causing the tonsils to be pushed down similar to that suggested for Chiari I malformation [150,151]("push" hypothesis, see Comment no. 14).

a) Is the concept of spinal cord abnormality testable ("pull" hypothesis)? [Moderator see [152]].

b) Are the 'morphological changes of cross-sectional shape and relative position of the cord' especially in the apical region not just adaptive to the deformity *from whatever cause*? (see Figure 2 and Comment no. 10, Response).

c) Is the concept of asynchronous nerve root growth testable? (see Comment no. 28, Response b).

d) In AIS subjects should *PCF *volume be measured in relation to each of the lower position of cerebellar tonsils, tonsillar ectopia, syringomyelia, abnormal SSEPs and the scoliosis? ("push" hypothesis)[150,151].

e) In any AIS subjects with *PCF hypoplasia*, should reciprocity be sought with supratentorial brain abnormalities? [[66-72], Moderator see [73,74]].

f) Do you relate the smaller skull vault posteriorly in AIS subjects [10] to *PCF hypoplasia?*

#### Response

a) This concept can be answered by a biomechanical model to test the biomechanical parameters of spinal cord and geometric modification of scoliotic spine. The project is now in progress.

b) Using the *MetaMorph *imaging system, we have observed that the cross-sectional shape of the cord was fairly symmetrical. Therefore the altered cross-sectional shape was unlikely related to compression force from the concavity of the spine, which should result in an asymmetrical shape of the cord (Moderator see Figure 2 and [2-4]).

c) We have not yet worked on this. Theoretically it should be feasible if a biomechanical model can be built.

d) Measurement of *PCF *volume is one of the objectives in our study on volumetric analysis of brain in AIS. This project is a joint collaboration with the Brain & Body Centre of Nottingham University, UK, supported by the British Scoliosis Research Foundation (Moderator, completed December 2007).

e) We think this is the correct approach though the supratentorial brain abnormality might only be detected by sophisticated volumetric analysis, not by conventional MR sequences.

f) Yes, both posterior skull vault and posterior cranial fossa are related to endochondral ossification, which is found to be uncoupled with membranous ossification in the vertebral column of AIS subjects.

### Comment no. 14

#### Posterior cranial fossa, Chiari I malformation, foramen magnum, and skull base abnormality

Chiari I malformation, with tonsillar descent <3 mm is associated in some patients with a *congenitally hypoplastic posterior fossa*: *reduced length of clivus, basisphenoid, basioccipu, increased angle of tentorium and CSF flow abnormalities *[151]. Neuroradiologists generally consider that tonsillar descent of up to 6 mm is not considered pathologic in subjects between 5–15 years [153], Jaspan T, personal communication].

In AIS subjects Cheng et al [32] redefined the MRI reference level to diagnose the asymptomatic Chiari I malformation as any inferior displacement of the cerebellar tonsil below the line at the foramen magnum connecting the *basion *and *opisthion*. Scoliosis severity correlated significantly with a lower position of the cerebellar tonsil.

Papers on skull morphometry in AIS girls have been presented recently by Dr Chu and colleagues: one paper [10] reports on the skull vault in 10 AIS patients compared with 10 healthy girls, and the latter [8] reports that:

"The length of the hypophyseal fossa was significantly shorter while the length between the dorsum sellae and basion was significantly longer in 28 AIS girls when compared to 18 age-matched controls based on midline sagittal MR image of the brain, indicating that abnormal growth probably affects the skull base."

a) Do you have any data on the dimensions of the posterior cranial fossa and foramen magnum for these AIS subjects compared with the normals?

b) Did any of the patients have a Chiari I maformation?

c) How do the findings of Yeung et al [8] showing skull base abnormality and disproportion In AIS girls relate to the Chiari 1 malformation and the postulated neural axis tether?

d) Do you have follow-up data on CSF motion at the cranio-cervical junction in AIS? [154].

#### Response

a) We found that the foramen magnum was significantly larger in AIS subjects than normal controls [35]. We have not yet looked into the dimensions of posterior cranial fossa but it would be an interesting area to explore.

b) None of the subjects included in the study has Chiari I malformation, syringomyelia or any cord abnormality (the presence of the above features are excluding criteria in this cohort).

c) Yeung et al [8] showed that the skull base geometry was different between AIS subjects and normal controls which could be related to uncoupled endochondral-membranous bone formation. Together with our recent finding of larger foramen magnum in AIS, these changes could be related to adaptation to low-lying cerebellar tonsils in AIS. The latter might be related to a neural axis tether at the distal end of the cord.

d) The study on CSF motion with larger sample size has been published [35].

### Comment no. 15

#### Embryology, neural crest and Chiari I malformation – mesodermal and occipital somites

A fundamental characteristic of the vertebrate body plan is segmentation along the anterior-posterior axis. Somite formation involves a molecular oscillator, the segmentation clock, in combination with gradients of signaling molecules and genes that regulate the bilaterally symmetry of somite formation [155]. According to O'Rahilly and Muller [156,157] in humans four occipital somites (identified in embryonic stage 13) and their sclerotomic material form two bilateral masses. The fourth sclerotome separates in stage 14 and develops like a vertebra. This and the remaining sclerotomic materal form the basiocciput and exoccipital parts of the chondrocranium [158].

#### Embryology of neck and shoulder

The research of Dr Chu and colleagues raises questions about the embryology of the neck and shoulder which, according to Matsuoka et al [75], has undergone a complex evolutionary history. The neural crest anchors the head onto the anterior lining of the shoulder girdle, while a Hox-gene-controlled mesoderm links trunk muscles to the posterior neck and shoulder region. Loss or dysplasia of the *post-otic neural crest *derived basicranial (clivus) bone attachments for the internal pharyngeal and laryngeal constrictors and the ensuing widening of the foramen magnum are the primary mechanical cause of the Arnold-Chiari I malformation. Le Douarin et al [159] note that the neural crest is strongly influenced by environmental cues and by Hox gene expression. Facial skeleton morphogenesis is under the control of a multistep cross-talk between the epithelium (endoderm and ectoderm) and neural crest cells.

#### Response

In a recent study, we have found a larger foramen magnum in AIS [35]. Together with our earlier finding of low-lying cerebellar tonsils in AIS [34], these features might suggest a dysfunction of neural crest during the embryogenesis. The vertebral deformity; however, is only manifested during puberty, the latter might be more dependent on postnatal factor(s) rather than embryonic somite formation.

## Motor control problem and brain asymmetries in AIS

### Comment no. 16

#### AIS as a motor control problem

Herman et al [66] found that processing of vestibular signals within the *CNS *yielded the highest correlation with curve magnitude. They considered that idiopathic scoliosis was a motor control problem. A higher level CNS disturbance was thought to be responsible for visuo-spatial perceptual impairment, motor adaptation and learning deficits; these lead to a recalibration of proprioceptive signals from the axial musculature causing idiopathic scoliosis. How can these findings be explained by tether of the spinal cord and brain stem? (Moderator: Veldhuizen et al [67] state that the most likely cause of idiopathic scoliosis involves an alteration of the motor drive at the spinal cord level, either from altered sensory input at the same level or from a central mechanism to produce an idiopathic scoliosis).

#### Response

Tether of spinal cord might be one component of the asynchronous neuro-osseous growth and we do not exclude the other important components in the neuro-osseous growth, which are the brain and skull. The cord tethering cannot fully explain the higher level *CNS *disturbance suggested by Herman and therefore we have looked at the volumetric regional brain volume difference between AIS subjects and normal controls [[71,72] Moderator see [73,74]].

### Comment no. 17

#### AIS and brain asymmetries

Dr Chu and colleagues have recently presented MRI evidence in AIS girls of abnormal asymmetric brain volumes [71-73]. Might such brain volume asymmetries in AIS be related to the spinal deformity in AIS?

#### Response

In the pilot study [71] comparing the regional brain volume in 20 AIS subjects and 20 age and sex matched controls, we identified a number of regional brain volume differences between the two groups which supports the hypothesis that AIS is a *systemic neuro-osseous disease*. In brief, significant unilateral regional differences were found in the following regions: left thalamus and left postcentral gyrus of AIS patients were significantly larger than the control subjects. Anterior and posterior limb of right internal capsule, right caudate nucleus, right cuneus and left middle occipital gyurs of AIS patients were significantly smaller than the control subjects. Some regions were bilaterally involved: Perirhinal and hippocampus regions were larger in AIS while inferior occipital gyrus and precuneus were smaller than the corresponding regions in the control subjects. In the midline, the volumes of corpus callosum and brain stem in AIS patients were significantly larger than the control subjects. The above findings suggest anatomic and likely functional imbalance of the descending corticospinal tracts as well as brain regions modulating motor function and coordination including deep nuclei, premotor cortices and midbrain structures. Asymmetries were also found in the temporal and occipital lobes which were involved in visual and spatial processing. Most importantly, the asymmetry of callosal commissural fibers generates the hypothesis that a primary deficit in interhemispheric coordination could play a role in the primary pathophysiology of the disorder [Moderator see [72,73]].

## Skull evolution and inactivated MYH16 gene in humans

### Comment no. 18

Stedman et al [76] formulated the hypothesis that a disabling mutation of the gene *MYH16 *produced a decrease in jaw-muscle size that removed a barrier to the gracile remodelling of the human cranium which consequently allowed an increase in the size of the brain. The hypothesis is controversial [160-164]. The *MYH16 *gene encodes the predominant myosin heavy chain, a critical protein component of sarcomeres, expressed in powerful masticatory muscles found in most primates. This gene is inactivated in humans. Stredman et al estimate that the *MYH16 *mutation as a coding sequence deletion appeared approximately 2.4 million years ago (*MYA*), predating the appearance of modern human body size and the emigration of *Homo *from Africa. Perry et al [163] date the deletion to 5.3 MYA. Because of -

a) the close parallel between neuro-cranial and neuro-spinal morphogenesis [24,134], and.

a) the low or nil prevalence of idiopathic scoliosis in non-human primates [165].

- is it reasonable to suggest that the inactivated *MYH16 *gene in humans may have relevance to the difference between humans and non-human primates with respect to -

a) spinal morphology [166], and

b) the prevalence of idiopathic scoliosis?

#### Response

We think it is a possibility.

## Immature scoliotic vertebral vascular biology and prognosis

### Comment no. 19

To our knowledge there are no reports of blood vessels supplying the growth plates of vertebral bodies in AIS. Mineiro [167] reported dilated vessels and vascular 'lakes' at each end of normal vertebral bodies from 9–13 years of age. It has been proposed [90] that dilated vessels and vascular 'lakes' adjacent to endplate physes of immature scoliotic vertebrae of living subjects might be sought by using gadolinium-enhanced MR imaging [88] and/or a serial study of diffusion characteristics in the spine after gadodiamide injection [89].

a) Can you apply such a radiological technique to the vertebrae of subjects with AIS?

b) Might the results of applying such techniques shed light on the mechanism of any uncoupled, or asynchronous, neuro-osseous growth?

c) May any positive findings with these radiological techniques in combination with the detection of:

i) platelet dysfunction [168,169], and/or

ii) endothelial-derived marker plasma proteins in response to physical exercise [170] and/or exposure to vibration [171] to enable the discrimination of progressive from non-progressive curves [90]?

#### Response

a) & b) We have not injected gadolinium contrast agent into our AIS subjects. However, from experience of imaging other pediatric patients, any "dilated vessels" and vascular 'lakes' adjacent to endplate physes of immature scoliotic vertebrae are probably too small to be resolved by MR imaging by a routine 1.5 Tesla machine. It might be possible if a more powerful MR magnet is used. However, the enhancement pattern of the endplates probably can be calculated by a time-intensity curve, which can indirectly reflect the vascularity of the endplates. If applicable, the results might reflect the vascularity of the endplates, which might indicate the growth potential of the immature spine.

c) It is difficult to predict at this juncture. We might need a large comparative study between AIS with progressive and non-progressive curves to reach a more definite conclusion.

## Systemic melatonin-signaling pathway dysfuntion in AIS

### Comment no. 20

In progressive AIS, Moreau et al [105] reported melatonin-signaling transduction to be impaired in vertebral osteoblasts, myoblasts and lymphocytes caused by the inactivation of Gi proteins [Moderator see [109]]. In 2006 their presentations showed this to be associated with high levels of a circulating protein P factor that appears essential for the initiation and progression of AIS through a specific signalling action during a postnatal window [107,108]. In 2007, Moreau et al [111] reported the use of lymphocytes to develop a functional blood assay for the early detection of AIS and for the identification of children at risk of curve progression; this test can be performed without any prior knowledge of mutations in any defective genes causing AIS.

A systemic abnormality of cell differentiation is proposed as a novel mechanism in the pathogenesis of AIS [106]. These findings suggest that the abnormality of AIS relates to the vertebrae rather than to the spinal cord and meninges. Considered with anthropometric findings for AIS showing widespread skeletal overgrowth [5,13-15] an abnormality of vertebral body growth plates (physes) is suggested that leads to skeletal overgrowth and growth conflicts [[172], Moderator see [103,173]].

#### Response

The mechanism proposed above is in agreement with our observation of abnormal lengthening of the vertebral column in AIS subjects. In our hypothesis, the initiating factor is vertebral column overgrowth while the spinal cord itself retains its usual growth rate and therefore lags behind resulting in relative shortening and tethering. The tethered cord itself might then act upon the spine to initiate the thoracic lordoscoliosis deformity (Figure 1).

## Points of controversy about the asynchronous neuro-osseous growth concept

### Comment no. 21

As a neuroradiologist I see many growing patients with proven spinal cord tethering, for instance by a thickened filim terminale or an intraspinal lipoma. Some develop neural disturbances but most do not have scoliosis and if so, often only a minimal scoliosis curve. Some fetuses develop spinal cord tethering again mostly without scoliosis. The suggestion that spinal cord tethering may of itself cause adolescent idiopathic scoliosis is theoretical. If so, why don't all patients with cord tethering develop scoliosis?

#### Response

Thank you for raising an interesting observation. In AIS, there is evidence from our group of abnormal systemic growth which include the appendiceal skeleton [5] and axial skeleton [6,7]. Our group has previously reported asynchronous growth of anterior spinal column with posterior column [6] and spinal cord [1]. Without the abnormal skeletal growth, the mere presence of spinal cord tethering may not necessarily result in scoliosis. This probably explains why infants with "congenital" type of cord tethering – like thickened filum terminale and intrathecal lipoma – do not develop significant scoliosis.

### Comment no. 22

This is a very interesting description of the deformity of vertebrae in adolescent idiopathic scoliosis. However, it is just that: a description. We know the vertebrae are deformed and advanced imaging technology permits better visualisation and measurement. Chu et al then argue that this deformity is its own cause: *because*, in AIS, there is a disproportion between spinal and cord length, *therefore *the deformity was caused by this disproportion. They have not demonstrated a cause-and-effect relationship, only an association. They have not examined whether this proposed pathogenesis agrees with clinical observation or basic anatomy. How can the spinal cord, which has the consistency of cold porridge, tether anything against the powerful force of growth? Surely far greater clinical signs of cord tension would bring the patient to medical attention before the scoliosis was detected?

Does the concept of uncoupled neuro-osseous growth exist outside of scoliosis studies? A search of *PubMed *suggests not. They may be quite correct in their speculation, but surely solid evidence is necessary before anyone starts de-tethering the spinal cords of every mild scoliosis that presents in the hope of preventing progression?

#### Response

In the study, we have demonstrated an association between disproportionate spine-cord length and scoliosis deformity. Based on the clinical observation of abnormal SSEPs which is frequently reported in AIS, we propose that the spinal cord is probably under tethering. However, as all of the AIS subjects in our study do not have clinically detectable neurological deficit, the proposed cord tethering should be subtle and hence we call it *subclinical tethering*. Though not causing functional disability, this subclinical tethering might be involved in the pathomechanism initiating the scoliosis deformity. Further study to test this hypothesis has been planned in collaboration with Professor Aubin's group in Montreal using a biomechanical model of the spinal cord as a mechanical tether.

### Comment no. 23

*Tether, thoracolumbar and lumbar scoliosis and curve laterality*.

a) If the neuraaxis tethers the anterior spinal column to create thoracic AIS how can it also determine thoracolumbar and lumbar curves?

b) If the neuraaxis tethers the anterior spinal column, how can this determine the laterality patterns of AIS?

#### Response

a) We do not have the answer to this question. So far we have studied AIS subjects with predominantly right thoracic curve. It might be interesting to look at those with a predominant lumbar curve.

b) In the recent published article by Kouwenhoven et al [57] in the normal adult non-scoliotic spine there is predominant vertebral axial rotation to the left of high thoracic vertebrae and to the right of mid- and lower-thoracic vertebrae which differs from an equal right-left distribution. It might be this pre-existent pattern of vertebral axial rotation that determines the most prevalent pattern in AIS.

### Comment no. 24

#### Tether, syringomyelia and curve laterality

Cord tethering and Chiari I malformation appear to be major causes of syringomyelia and scoliosis. The laterality of these curves is reported to be about equal, left and right [77]. If cord tethering in syringomyelia does not cause a predominant laterality for the associated scoliosis, how can a short spinal cord explain the laterality patterns of AIS?

#### Response

In those cases with structural cord tethering and syringomyelia resulting in scoliosis, they are not regarded as idiopathic scoliosis. Also in our group, we have observed the presence of a wider foramen magnum [35] which is contrast to the reported typical cases of Chiari I malformation manifested with juvenile idiopathic scoliosis or syringomyelia in subjects with neurological manifestations. We explain the predominant thoracic AIS curve right laterality by the pre-existent normal axial rotation [57] as stated in the response to Comment no. 23.

### Comment no. 25

#### Tether, normal vertebral growth and neck flexion

The normal upper spinal cord allows excursion of up to 18 mm [16,28]. If the spinal cord tethers the anterior spinal column why is there not restriction of neck flexion in AIS patients?

#### Response

It may be due to the relative elasticity of the cord and that it is still within the subclinical range. In future if we can do dynamic MRI imaging in the standing position, we may be able to analyse such phenomenon in more detail.

### Comment no. 26

#### Tether, scoliosis and restricted neck flexion

Floman [64,65] reported a distinct type of 'idiopathic' thoracic scoliosis in 12 adolescent male patients accompanied by a striking restriction of neck flexion, in 9 with mild thoracic or cervical spine pain. The scolioses were right convex in 10 and left convex in two. It was thought possible [65] that a *short spinal cord *may be the underlying cause for both the thoracic scoliosis and limited neck flexion. Do you have observations on neck flexion in the subjects?

#### Response

We did not observe any significant restriction of neck flexion in this cohort.

### Comment no. 27

Have the investigators addressed the mechanical properties of the spinal cord relative to the physiological forces? If the hypothesis is that the cord mechanically resists, in part, the growth of the vertebral column, how much tension would the cord need to generate to affect the growth of the spine? Is there evidence to suggest that the tensile stiffness and strength of the cord, along its length as well as at the cranial and caudal boundaries, are sufficient to resist those loads?

#### Response

The above questions can be answered by a biomechanical model of the spinal cord. We are collaborating with Professor Aubin in Montreal to work on this project and are currently applying for funding.

### Comment no. 28

To explain the extra-spinal left-right skeletal length asymmetries associated with AIS you speculate that the "....observations about asymmetry of skeletal growth might be part of the component in the process of *asynchronous neuro-osseous growth *in the pathogenesis of AIS." (see Response to Comment no. 34).

a) You invoke a new speculation namely '*asynchronous neuro-osseous growth'*. Does this differ from the Porter concept of *'uncoupled neuro-osseous growth' *[28] with vertebral growth tethered by spinal cord growth? If so, could you please define '*asynchronous neuro-osseous growth'*.

b) How do you explain the extra-spinal left-right skeletal length asymmetries of AIS by '*asychronous neuro-osseous growth'*? Are you suggesting that some nerve roots and peripheral nerves develop left-right asymmetry in adolescent growth which in some way causes certain long bones to grow asymmetrically in AIS? In this connection Roth [23] concluded that the behaviour of the spinal cord and nerves in scoliosis suggests 'a *primary growth insufficiency of the cord or the nerves*, *or of both'*, which account for the curvatures of the spine. Roth [23] wrote: "In other words, the spine cannot grow straight but is forced into curvatures." Some support for this view comes from Repko et al [91] who find that spinal peripheral nerves from the convexity and concavity of 9 patients with idiopathic scoliosis have morphological changes in myelin sheaths and axons compared with 2 patients without scoliosis that may help in the search for the pathogenesis of idiopathic scoliosis [Moderator see [92]].

c) Might any asymmetries of rib [80,112], upper arm [13,14,78,79,81] and tibial length [82,174] in AIS be explained by such left-right asymmetries of nerve roots and peripheral nerves?

d) If so, how would you account for the iliac length asymmetry [82] associated with each of lumbar and thoracolumbar AIS?

#### Response

a) Our hypothesis [1,2] is probably similar to Porter's [28] concept. Both our concepts indicate a disorder in differential growth between the skeletal system and nervous system. We use *'asynchronous****' ***to mean the pace of the growth is now disturbed, while the word *"uncoupled" *means there is a disruption of previously coupled growth. We think *'asynchronous' *is probably a more objective term because we cannot be certain whether the growth of nervous system and skeletal system were *'coupled' *in AIS before the onset of the disorder.

b) We cannot be certain about the whether the nerve roots [23] and peripheral nerves [91] develop left-right asymmetry as anatomically no imaging technique now available can answer this question [Moderator see [92]]. What we mean is that both the skeletal system and nervous system are involved in the development in AIS of left-right skeletal length asymmetry of the appendicular skeleton as part of the subset of abnormal skeletal growth within the whole set of *'asynchronous neuro-osseous growth'*.

c) It is possible but cannot be proved by radiological means currently. It seems that Repko et al [91] provide some histological support for this view.

d) The iliac length asymmetry [82] might be a part of the subset of abnormal skeletal growth in AIS.

### Comment no. 29

In connection with their *uncoupled, or asynchronous, neuro-osseous growth concept *would Dr Chu and colleagues respond to the following comments:

a) the view that pontine and hindbrain regions are the likely sites of primary pathology that could lead to idiopathic scoliosis [94],

b) speculation that a process producing left-right spinal asymmetry (? from end-plate physes, osseous, discal, or neuromuscular tissues) together with -

i) rapid spinal elongation

ii) brain maturation,

iii) upright posture and

iv) trunk movements -

may contribute to the initiation and progression of AIS [[71,85], Moderator see [86,87]](see Responses to Comments nos. 16 & 17).

c) anecdotal evidence that prolonged rest in bed halts the progress of scoliosis [95,96]. In over 30 patients in whom scoliosis was advancing, Cobb kept children in bed for 22 hours per day; except for one patient no progress was noted after three months.

#### Response

a) The asynchronous neuro-osseous growth concept accommodates Lowe's hypothesis [94] of pontine and hindbrain regions to be a likely site of primary pathology. As our group have shown changes in the posterior skull vault and foramen magnum in AIS [10,35], there might also be changes in the corresponding brain structures that are housed in the above region. We plan to investigate this aspect in our new volumetric brain analysis study [Moderator, see [71-74]].

b) Asynchronous neuro-osseous growth accommodates the left-right spinal asymmetry concept which involves osseous growth and neuromuscular co-ordination.

c) We speculate that rest in bed [95,96] might also halt the tethering effect on the cord but the hypothesis is again better tested by the biomechanical modelling.

## Relative anterior spinal overgrowth (*RASO*) concept

### Comment no. 30

The papers of Guo et al [6,7] address relative anterior spinal overgrowth and AIS in which the Roth-Porter hypothesis of *uncoupled neuro-osseous growth *was considered, but rejected. This was because knowledge of normal vertebral growth supports the view that the scoliosis deformity in AIS is related to longitudinal *vertebral body growth *rather than growth of the *vertebral canal*. As pathomechanism they adopted the concept of *primary skeletal change *affecting the sagittal plane of the spine, and in the pathogenesis they proposed a novel histogenetic hypothesis of *uncoupled endochondral-membranous bone formation*. The latter was viewed as part of an *'intrinsic abnormality of skeletal growth in patients with AIS which may be genetic.' *The authors of the current paper state that the findings support the concept of *uncoupled neuro-osseous growth *in the pathogenesis of AIS [28].

a) Do Dr Chu and colleagues reject the *primary RASO hypothesis *of *uncoupled endochondral- membranous bone formation in AIS pathogenesis?*

b) Does *RASO *now provide a biological mechanism for a *'maladaptation of the growing immature spine to a tether created by a relatively short spinal cord'*?

#### Response

a) No, we think the hypothesis of *uncoupled endochondral-membranous bone formation *in AIS pathogenesis [6,7] can co-exist with the current concept of *uncoupled neuro-osseous growth *[1,2]. They are probably different components of the same systemic abnormality of scoliosis.

b) We think *RASO *is a contributory factor in the biological mechanism for a maladaptation of the growing immature spine.

## Thoracospinal concept

### Comment no. 31

At the 2006 IRSSD meeting I presented the argument for girls with the condition of right thoracic adolescent scoliosis to be no longer termed idiopathic [114]. These girls form a sub-group of AIS within the *thoraco-spinal concept of the pathogenesis *for which I proposed the acronym *Rcx-T-F-AS *[80,112]. The evidence for defining this sub-group separate from AIS is anthropometric, experimental and biomechanical. The features of this entity include: laterality and pattern of curve, height, weight, gender, menarche, sympathetic dysfunction, abnormalities of muscle fibres and platelets, and osteoporosis [[113,114], Moderator see [92,115]].

Our observations together with known and well-documented specific combination of morphological manifestations of the somatic deformity and physiological characteristics of the patients strengthen the recently propounded view that the right thoracic adolescent scoliosis in girls *(Rcx-T-F-AS) *is not just a spinal deformity but a neglected *paediatric nosological entity *probably with related extra-vertebral influences [114]. We do not relate these observations to other types of adolescent scoliosis, i.e. *Rcx *in boys, or *Lcx *in girls and boys.

#### Response

We agree that right thoracic adolescent scoliosis in girls might be a subset of AIS and we are working on the hypothesis of asynchronous neuro-osseous growth as the pathogenesis of this group of patients whom probably should not be named "idiopathic". It would be an interesting idea to compare right thoracic scoliosis in boys and subjects with left thoracic scoliosis; however these cases form a minority group of scoliosis subjects in our centre and we have not yet reached enough sample size to perform a case control comparison [Moderator see [74]].

### Comment no. 32

In the response to Comment no. 31 the authors agree that right convex thoracic adolescent scoliosis in females (*Rcx-T-F-AS*) may be a subset of AIS which "....probably should not be named 'idiopathic"'. Patients with this type of deformity comprise about 80 per cent of those referred to hospital with AIS apart from two or three small subset. How does your proposed pathogenetic hypothesis of '*asynchronous neuro-osseous growth' *explain -

a) the complex of morphological and pathophysiological manifestations of Rcx-T-F-AS [113,114], and

b) the simultaneous 3-D vertebral translation?

#### Response

a) The *hypothesis of asynchronous neuro-osseous growth *proposes that during the rapid puberty growth of the adolescent girls, skeletal growth is faster than spinal cord growth; therefore they are asynchronous and therefore a tether is present. As there might be a pre-existing trend of thoracic vertebral axial rotation towards the right side [57] the faster growth of thoracic spine might form a right convex curve morphologically. While physiologically, as the cord is 'tethered', this gives rise to abnormalities in somatosensory evoked potentials (SSEPs). We think the *asynchronous neuro-osseous growth *is one of the important components for *Rcx-T-F-AS *and might not fully explain the whole spectrum of its complexity.

b) Simultaneous 3-D vertebral translation is better answered by a mechanical model development and simulation of the spinal cord as a mechanical tether. We are currently collaborating with the Montreal group to work in this direction.

## Origin in contracture at the hips

### Comment no. 33

I cannot accept this hypothesis of uncoupled neuro-osseous growth namely that: *"Such tethering could affect anterior spinal column growth leading to a progressive hypokyphosis and lordosis of the thoracic spine which coupled with the subtle neurological dysfunction can then initiate the process of lordoscoliosis deformity in the thoracic spine."*

If a shortened spinal cord influences vertebral spinal growth in idiopathic scoliosis the neurological dysfunction cannot be subtle. My explanation for the initiation of the lordoscoliosis of the thoracic spine in idiopathic scoliosis is strictly biomechanical [[116-121], Moderator see [122,123]]. The origin lies in the *'syndrome of contractures' *resulting from the left-sidedness of normal fetal positioning [121]. This syndrome includes abductor muscle contracture of the right hip which leads to movement asymmetry of both hips transmitting asymmetrical loading from the 'missing' (restricted) movements of the right hip to the pelvis and spine. This produces rotational deformity of the spine with stiffness, and in some children a lordotic deformity, of the thoracic spine, making the anterior spinal column longer than the posterior spinal column (1^st ^etiological group of scoliosis [120]). Hence, vertebral column deformity develops first, and relative shortening of the spinal cord occurs secondarily.

#### Response

We do not exclude the biomechanical hypothesis in the pathogenesis of AIS. Our hypothesis agrees with the conclusion that there is initial overgrowth of the spinal column while the spinal cord fails to keep in pace with vertebral growth and therefore results in secondary relative shortening. We extend the above observation and suggest that the tethered cord might play an enhancing role in the development of lordoscoliosis by creating the tether.

## Neurodevelopmental concept

### Comment no. 34

We identified skeletal growth abnormalities thought to be related to AIS pathogenesis and incorporated them into the *Nottingham concept of pathogenesis *[[175], Moderator see [85-87]]. These include: a) widespread skeletal overgrowth [12-14,78,176]; b) extra-spinal left-right skeletal length asymmetries [[13,14,78-83,174,176,177], Moderator see [178,179]]; c) proximo-distal lower limb skeletal length disproportion [[83,174], Moderator see also [177,179]]; and d) other skeletal disproportions [12]. A novel neurodevelopmental mechanism was proposed for AIS pathogenesis [[85], Moderator see [86,87]].

In connection with the *widespread skeletal overgrowth*, Cole [13,14,176] found an increase in each of 17 anthropometric components in 66 preoperative AIS girls compared with 693 age-matched healthy girl (5 unpaired and 12 paired components]; the regions included stature and sitting height (each corrected for Cobb angle), subischial height, biacromial width, biiliac width, total arm lengths, upper arm lengths, forearm-with-hand lengths, total leg lengths, tibial lengths and feet. There is controversy about overgrowth in relation to stature and AIS [14,132]. The skeletal overgrowth in AIS is more evident in younger than older subjects as judged by ranking in centiles, standard deviation scores and bone ageing [13,14,174,179,180].

It is not known whether the extra-spinal left-right skeletal length asymmetries and skeletal disproportions signify any local involvement in the spine. We speculate that they do [82,83,85] as for the general skeletal overgrowth [[6,7], Moderator see [86,87,173]]. In this connection there is indirect evidence suggesting that in *idiopathic *scoliosis both the *hypokyphotic *and *axial rotation components *about the apex of the deformity may be determined by local processes in the spine not present in neurogenic scolioses [93,124-127]. The *CNS *component of our neurodevelopmental concept [85] is speculative but accords with evidence that the pontine and hindbrain regions are the likely sites of primary pathology that could lead to idiopathic scoliosis [94]. The initiating scoliogenic mechanisms in the trunk are unknown but may involve axial vertebral rotation [22,57,181], ribcage [15,80,112-114,182-186] and trunk movements [[84,85], Moderator see [86,87,173,187-190] for the relation of skeletal maturity to curve progression see [191,192]].

a) How do Dr Chu and colleagues account for the association between thoracic apical rotation and upper arm length asymmetry in thoracic AIS subjects [81]?

b) How does the uncoupled, or asynchronous, neuro-osseous growth concept explain the other patterns of extra-spinal left-right skeletal length asymmetries with AIS?

#### Response

a) We have not looked into details of upper arm length asymmetry and thoracic apical rotation in the previous database but we will take into account the above interesting observations in our ongoing work. We speculate that all the above observations about skeletal growth asymmetry might be part of the component in the process of asynchronous neuro-osseous growth in the pathogenesis of AIS

b) As mentioned in the previous point, we cannot explain other patterns of extra-spinal left-right skeletal length asymmetry at the moment but we speculate that all the above observations might be part of the whole process of asynchronous neuro-osseous growth in the pathogenesis of AIS

## Biomechanical growth modulation concept

### Comment no. 35

In a recent electronic focus group, Dr Stokes [103] addressed the concept of biomechanical spinal growth modulation in the pathogenesis of progressive AIS [Moderator see [104]]. His *'vicious cycle' *pathogenetic hypothesis requires a pre-existing scoliosis curve that initiates asymmetric muscle loading on vertebral bodies which in turn causes worsening of the scoliosis, while everything else is anatomically and physiologically normal. How does your uncoupled, or asynchronous, neuro-osseous growth concept relate to biomechanical growth modulation concept proposed by Dr Stokes?

#### Response

Our hypothesis of asynchronous neuro-osseous growth proposes that the relative short cord can lead to hypokyphosis and hence curve initiation with or without progression of the scoliosis. Therefore, it might be the starting point as well as an aggravating factor in the 'vicious cycle' hypothesis of Dr Stokes [103,104].

## Authors' contributions

WC with her colleagues in Hong Kong, WL, B Ng, TL, KL, XG and JC wrote the Statement based on their published research findings and also the responses to each Comment, PD was the Moderator communicating electronically with all IBSE members some of whom provided the Comments, GB with PD wrote the Abstract and Background, structured the text and compiled the list of references with TJ providing them with neuroradiological expertise. All authors read and approved the final manuscript.
